# Pelvic Peritonitis

**Published:** 1888-06

**Authors:** W. C. Fisher

**Affiliations:** Galveston


DANIEL'S

### Texas Medical Journal.

Published Monthly.—Subscription $2.00 a Year.

Vol. 3. AUSTIN, JUNE, 1888. No. 12.

By unanimous vote of the members, this Journal was made the Official Organ of the Austin District Medical Society.

### Original Articles.

CONTRIBUTED EXCLUSIVELY TO THIS JOURNAL.

*The Articles in this Department are accepted and published with the understanding that we are not responsible for, nor do we indorse the views and opinions of the writers by so doing.*

### PELVIC PERITONITIS.

*By W. C. Fisher, M. D.*

*A paper read before the Galveston County Medical Club, and contributed to Daniel's Texas Medical Journal.

*Mr. President and Gentlemen :*

IT is almost a matter of impossibility for us in this progressive age to select a subject that has not been thoroughly ventilated by the leading lights in the profession, or to introduce anything new and original; and I am merely sketching this for the purpose of hearing the members of our honorable club give us their views, and the benefit of their experience.

Pelvic Peritonitis may be defined as an inflammation involving the peritoneum of the pelvic cavity, and like all inflammations has three stages; first, the stage of congestion, when there is increased heat, blood stasis and pain; the second stage, when there is the formation of lymph ; and third, where the inflammatory process goes on to the formation of pus.

It is possible in some cases, where the disease is recognized in the first stage, to limit it to this stage, but unfortunately such cases are rare, owing either to the patient applying to some old woman, or friend, for remedies, and not sending for the physician, until the disease has advanced to the second stage, or to the physician failing to recognize the case in this early stage. After the formation of lymph has taken place, our patient is in a critical condition, and it will require the most judicious treatment to prevent either death from high temperature or exhaustion, or to prevent the formation of pus; which is the most formidable stage of disease that we have to encounter, for unless this pus has an exit either by the surgeon's hand or by Dame Nature making an outlet, or, unless the pus become encysted, we are bound to have death sooner or later, from either acute or chronic septicæmia or pyæmia. And, even if the pus become encysted, we are in constant dread of the cyst bursting and relighting, so to speak, a smothered or dormant peritonitis, or the end may be still more rapid by the *shock* which often attends the bursting of these pus sacs.

From the intimate relation that exists between the peritoneum and the female organs of gestation and the cellular tissue that unites them, we can readily see the amount of damage that may be had is often the result of a severe inflammation of either the second or third stage, or both, and for this reason it behooves us to study carefully the causes that lead to this trouble, and, as far as in our power, eliminate them.

We are well aware that peritonitis is often the result either of an extension by contiguity of the inflammatory process from the cellular tissue, or the relation between the two is so intimate that they are often attacked at the same time.

Some authors are inclined to classify the two under a common name ; but the weight of authority is in favor of differentiating them on the ground that the two tissues involved are so entirely dissimilar, histologically.

Almost any factor that is capable of producing one of these diseases is equally liable to produce the other; that is, in a general way. I will dwell more particularly on several of the most common factors, and then mention the others in passing.

The ignorance that most young ladies have, regarding the menstrual function is often the cause of a severe attack of peritonitis, which if it does not end their earthly career, may, by the adhesions and bands of inflammatory lymph which remain, deprive them of that which, to a noble woman, is the greatest gift of God, namely, maternity; or if it is not so serious as this, they may be invalids for life, and unable to enjoy the pleasures and attributes of married life. Knowing the evil resulting from this ignorance, it is our duty, as family physicians, to tell mothers and those entrusted with the care of their daughters, of the importance of a certain amount of knowledge regarding this physiological process.

How imprudence during this time may produce so much trouble, is too well known to all of us for me to dwell on.

Another prolific cause is *abortion*, which, alas! too often meets the physician of the day, and with it we are confronted with the painful knowledge that the blood of the unborn infant has decreased in value in modern times. We do not meet these abortions alone in women of low moral standing, or in girls, the victims of misplaced confidence, who will resort to every means to hide their shame, but often in women whose standing is high, and whose moral characters are considered above reproach, and their only reason is because the demands of society are such that it is *distasteful* and *unfashionable* to have more than one or two children. All kinds of means are resorted to, both therapeutical and mechanical, to bring about this end. Every town and city of the day possesses either low old women, or doctoresses who are willing to produce abortions, or some physicians who prefer to make a living by murdering the unborn infant, to following their legitimate calling.

The perpetrators of such heinous crimes will not be found in the ranks of our honored fellows. Where they are to be found must be the work of the detective, and not the doctor.

Some young women are ignorant of the crime that they are co[illegible]-mitting, and are merely following in the footsteps of some of th[illegible]r seniors. A word of advice in this direction from the doctor, w[illegible]l often prevent a repetition. Hence, it is our duty to warn all, [illegible]

only of the great moral wrong they are committing, but also the great danger that their physical condition is subjected to.

Another potent cause is gonorrhœa, which is, as we all know, a very common affection with loose women, first affecting the vagina, thence the uterine cavity, mucous membrane of the tubes, and then the peritoneum. In all obscure cases, not only in those who we have every reason to believe are suffering from this complaint, but in those who are in the most refined circles, we should enquire very minutely into the history of the case; for all married men are not particularly scrupulous regarding the marital vow, and occasionally infect their wives; and, on the other hand, though not so often, some married women are equally regardless of their duty to their husbands, and become infected.

The other causes may be enumerated as surgical operations on the pelvic vicera, parturition, inflammation of the lining membrane of the uterus, injection of fluid into the uterus, traumatic inflammation, uterine displacement, etc.

The symptoms of this disease are well known to all of you, and I will merely mention them in the order given in Thomas' work, 'Diseases of Women: Pelvic pain and tenderness, sometimes great vesical irritation, usually increased thermometric range, nausea and vomiting, anxious faces, mental disturbance and tympanites.

I would call especial attention to the fact that we quite often meet a severe attack of this disease, when there is no exaltation of the temperature, but, on the other hand, rather a tendency to a sub-normal condition. This is mentioned in Thomas' work, and he says that to him who implicitly trusts to the revelations of the thermometer it will prove an unreliable guide, etc.

This has been my experience in several cases, in the past two years. (Some might say it was due to my thermometer, but as it was thoroughly tested and tried, that can be eliminated.)

The length of this paper is already beyond my intention, and instead of entering into description of the physical signs, I will refer to the works on genæcology which are numerous and most excellent. I cannot close, however, without making some remarks on treatment, and refering to a case or two in practice.

The sheet anchor in this disease is undoubtedly opium in some form or other, for it brings about both physical and physiological rest, which is the *sine qua non.* It must be given for its effect, and not according to the dose in our posological tables; it it simply wonderful what large amounts can be administered before we get the desired effect. The next indication is to reduce the high temperature which generally plays a very prominent part, and for this, nothing in my opinion, is equal to large doses of antipyrine, guarded by either digitalis or strapanthus. I formerly gave quinine in large doses for its antipyritic effect, but since the above remedy has been brought into such common use, I use it in two three grain doses, every three or four hours, as a heart tonic. I use turpentine stupes freely to the abdomen for the distressing tympanites, and in small doses, say five or ten minims, every four hours internally. I have never used it in the large doses recommended for diphtheria, but think, in the septic form, it would be good treatment, and I intend trying it in the future. Occasionally the tympanites will be so distressing that aspiration will be necessary, but this is indeed very seldom. In addition to the above remedies a large enema of castor oil and turpentine will often produce charming results. I have no experience whatever with the treatment of this disease by the "salines," but such treatment is endorsed by high authority and must have merit. It seems to me, that it would be very useful in the first stage, by relieving the full and congested blood vessels, and thus relieve tension.

If the peritonitis has become suppurative, and we can locate the pus, and it is doing damage, then we must give it an outlet either by the knife or an aspirator; but if we are in doubt, or if we have reason to believe that the pus has become encysted, then it is, in my opinion, best to wait, and build our patient up on tonics, good nourishing diet, plenty of fresh air and sunshine and cheerful surroundings. Always, though, warn our patient of the presence of the pus, so that she may be prepared for any accident that might occur.

If there is evidence of sepsis slowly killing our patient, we should advise laparatomy, if it offer any hope of recovery; and from the moribund cases reported by Mr. Lawson Tate, saved by this proceedure, we can afford to be bold. We must remember, though, that there are few Taits, and it will not do for us to immitate him too closely. It is often a very hard matter to decide just when to operate.

Several months ago I treated a very prominent colored nurse in this city, for a violent attack of peritonitis, which became suppurative. She developed a low form of septicæmia, and after consultation with four or five of our leading physicians, we came to the conclusion that the chances were very much against her, but that laparatomy would give her a chance. We decided to watch and wait twenty-four hours, and well we did, for in a few hours there was an escape of pus high up in the vagina and our patient made a good recovery. Perhaps, if the pus had delayed making its appearance at this opportune time, we would have had another death to record from laparatomy for suppurative peritonitis.

