# Errata

**Published:** 1888-06

**Authors:** 


ERRATA.—On page 506, fifth line from bottom, in Dr. Fisher's article, for "genarcology," read "gynæcology."

